# Supplementary material for: An advanced reliability reserve incentivizes flexibility investments while safeguarding the electricity market
Source: iScience. 2026 Jun 3;29(6):116176. doi: 10.1016/j.isci.2026.116176 (PMC13255031; doi:10.1016/j.isci.2026.116176)
Supplement: Document S1. Figures S1–S7, Tables S1–S3 [file mmc1.pdf]

iScience, Volume *29*

Supplemental information

An advanced reliability reserve incentivizes flexibility investments while safeguarding the electricity market

Franziska Klaucke, Karsten Neuhoff, Alexander Roth, Wolf-Peter Schill, and Leon Stolle

Supplemental information

S1. Input data for the capacity expansion model

In this section, we give an overview of the most important input parameters for the capacity expansion model.

Table S1: Capacity and cost assumptions, related to STAR methods

	**Capacity**	**Lifetime**	**Overnight costs**	**Fixed costs**	**Efficiency**	**Carbon content**	**Fuel costs**	**Var. costs storing in/out**
	GW	years	euro/kW	euro/kW	%	t/MWh$_{th}$	euro/MWh	euro/MWh
Run-of-river hydro	3.93	50	3000	30	0.9	0	0	-
Natural gas (CCGT)	0.00–∞	25	800	20	0.54	0.201	26.03	-
Natural gas (OCGT)	0.00–∞	25	400	15	0.4	0.201	26.03	-
Oil	2.82	25	400	7	0.35	0.266	41.65	-
Bio energy	11.06	30	1951	100	0.49	0	10	-
Onshore wind	115.00	25	1182	35	1	0	0	-
Offshore wind	30.00	25	3935	100	1	0	0	-
Solar PV	215.00	25	600	25	1	0	0	-
Lithium-ion batteries		20						
... power in/out	0–∞/0–∞		50/0	0.1/0	0.97/0.97	-	-	0.3/0.3
... energy [GWh]	0–∞		300	0.7	0.999989	-	-	-
Power-to-gas-to-power		22.5						
... power in/out	0–∞/0–∞		305/850	0/0	0.73/0.6	-	-	1.2/1.2
... energy [GWh]	0–∞		2	0	1	-	-	-
Open pumped hydro storage		80						
... power in/out	1.86/2.14		550/550	0/0	0.97/0.91	-	-	0.56/0.56
... energy [GWh]	471.23		10	0	0.999995	-	-	-
Closed pumped hydro storage		80						
... power in/out	6.56/6.41		550/550	0/0	0.97/0.91	-	-	0.56/0.56
... energy [GWh]	391.58		10	0	0.999995	-	-	-
Reservoirs		50						
... power out	0.82		200	30	0.95	-	-	0.1
... energy [GWh]	237.22		10	0	1	-	-	-

Notes: In the column capacity, if two numbers are connected with a hyphen, the model can choose endogenously in that range; otherwise, the value is fixed. The assumed carbon price is 130 euro/ton.

S2. Assumptions and database for demand-side flexibility options

S2.1. Demand response potential in energy-intensive industry

The following assumptions were taken as a basis for forecasting the future flexibility potential of energy-intensive industry:

1. Demand response in the form of load relinquishment is considered for the electric steel and aluminum industries due to their technical process conditions; load shifting is assumed for all other processes due to the high costs of lost production.
2. When differing load shifting potentials were reported in the literature, the maximum technically feasible potential has been used.
3. If no other information is available, it is assumed that the load flexibility potential of a process is $10\,\%$ of its installed capacity.
4. The installed electrical load of non-specific energy-intensive industries is based on BAFA data.[1] The values have been adjusted by the considered electrical load of the separately analyzed industries and those industries that are not suitable for demand response (e.g. electrified public transport).
5. According to a BCG study[2], the electricity demand of the non-specific energy-intensive industries will increase by $39\,\%$ until 2030 due to the increasing electrification of industry.
6. Due to the lack of detailed data in the literature on the duration of load change, it is assumed that the load flexibility potentials are available proportionally for $3\,\mathrm{h}$, $12\,\mathrm{h}$, $72\,\mathrm{h}$ and $336\,\mathrm{h}$. The maximum available duration of load change has been determined according to their technical requirements.

The basis of calculations, including references, is shown in table S2.

Figure S1 shows the projected flexibility potentials of industrial energy-intensive processes for different load change durations for 2030.

Integrating demand response into the production process requires initial investments and incurs both variable and fixed costs.[12] To model these costs, the following simplifying assumptions are made:

7. Only the cost of activating the load change is considered as a variable cost. Variable costs resulting from the duration of the load change are neglected.
8. The variable cost of the load change increases with the level of activated load. For simplicity, lower costs are considered for the first $20\,\%$ of available flexible load. This also reflects the wide range of values reported in the literature.

Table S2: Data and references on the demand response potentials of energy-intensive industrial processes, related to STAR methods

**Energy-intensive industrial process**	**Demand response potential** % of the installed load	**Installed capacity Germany** MW_{el}	**Max. duration of load change**[a] h	**Min. load change costs**[b] euro/MW_{el}	**Load change costs** euro/MW_{el}
Electric arc furnace	99[c]	1097[d]	336		283[e]
Aluminum	95[f]	543[d]	336		27[e]
Paper	95[g]	312[d]	12	10[d]	69[h]
Cement	90[i]	360[d]	336	80[j]	320[e]
Chlor-alkali process	54[k]	1484[l]	72	0[d]	100[l]
Air separation	70[m]	570[l]	72	0[n]	243[m]
Other	10[o]	1050[p]	336	10[n]	300[n]

[a]see assumption 6, [b]see assumption 8, [c] [3], [d] [4], [e]See variable load changing cost estimation calculations (equation 1), [f] [5], [g] [6], [h] [7], [i] [8], [j] assumption 25 % of the load change costs, [k] [9], [l] [10], [m] [11], [n]own assumption, [o]see assumption 3, [p]see assumption 4 and 5.

9. There are no storage losses in product storage.
10. By 2030, it is projected that existing production capacities will be underutilized as a result of the increasing relevance of the circular economy. The resulting surplus capacity can be employed for demand response overcapacity, thereby obviating the necessity for further investment costs for additional capacity expansion.
11. Investment costs for product storage depend significantly on product properties (e.g. density, state of aggregation, hazard potential, storage conditions, etc.) and the individual site conditions of production. Therefore, the investment costs for product storage for 1,2-dichloroethane (DCE) are assumed here for all processes for the purposes of simplification. 1,2-Dichloroethane is liquid under ambient conditions and has good properties regarding storability. It is stored in a coated vessel tank.[13] The specific investment costs for these storage systems are estimated at $5240\,\mathrm{euro/MW_{el}}$.[9]
12. Fixed costs of storage are neglected.

The variable load change costs in euro/MW for chlor-alkali process, paper, and air separation are determined by utilizing literature data (see table S2). However, given the absence of plausible values for electric steel, aluminum electrolysis and cement, their costs were estimated by calculating the revenue requirement. In this context, this value describes the costs to be covered in case of non-production due to load reduction. It is using the gross value added based on the work of Hourcade et al.[14] The revenue requirement (RR_x) for the process x is calculated using the product-specific gross value added (GVA_x), the product market prices

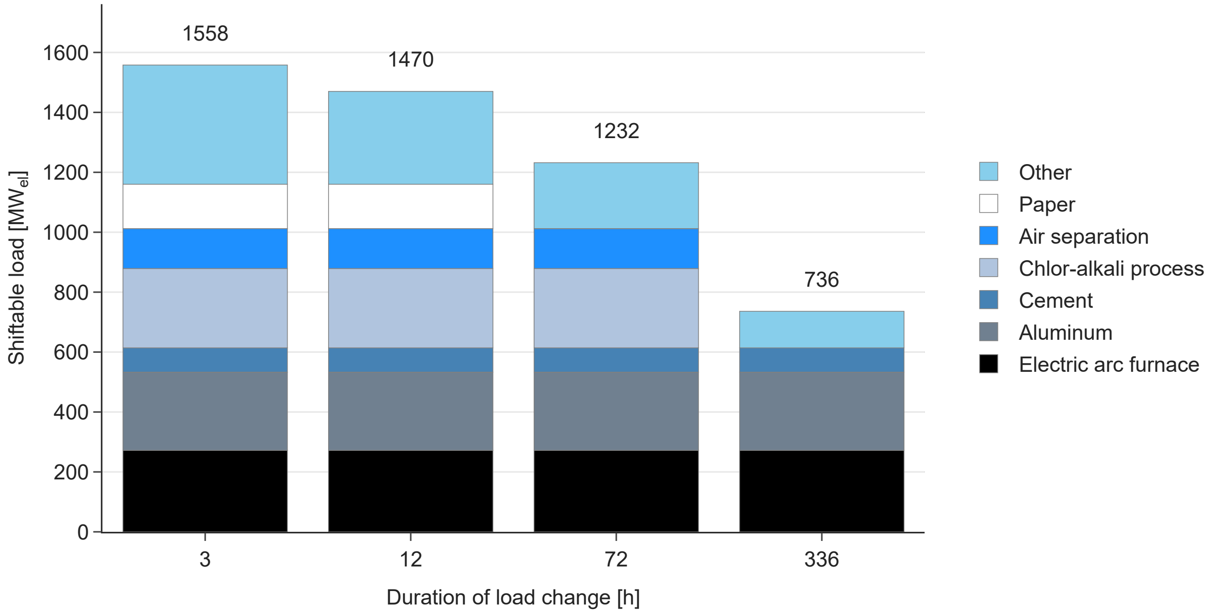

Figure S1: Projected potentials of flexible load of industrial energy-intensive processes for the year 2030 per available duration of load change

(p_{x}) and the product-specific electricity demand (e_{x}):

$$RR_{\mathrm{x}} = \frac{GVA_{\mathrm{x}} \cdot p_{\mathrm{x}}}{e_{\mathrm{x}}} \tag{1}$$

Table S3 provides the revenue requirement and the input data for its calculation and table S2 shows the estimated load change costs.

Table S3: Input data for calculating the costs of the load request based on the revenue requirement, related to STAR methods

**Energy-intensive industrial process**	**Product-specific gross value**[a] %	**Product market prices** euro/t	**Product-specific electricity demand**[b] MW_{el}/t	**Revenue requirement** euro/MWh_{el}
Electric arc furnace	25 %	600[c]	0.53	283
Aluminum	20 %	2000[d]	15	27
Cement	40 %	80[e]	0.1	320

a [14], b [4], c [15], d [16], e [17]

S2.2. Demand response potential process heat

The assessment of the load-side flexibility potential for process heat for the year 2030 based on data by the German Environment Agency [18], and was conducted under the following technical assumptions:

13. The provision of process heat is facilitated by electricity, given the availability of the requisite temper-

ature levels and process requirements. This is achieved through the utilization of a high-temperature heat pump, capable of reaching temperatures up to $160\,^{\circ}\mathrm{C}$, and a resistance heater, which operates within the temperature range of $200-500\,^{\circ}\mathrm{C}$.[19]

14. The average coefficient of performance (COP) of the high temperature heat-pump is 3.7[19] and the efficiency factor of the resistance heater η_{RH} is $99\,\%$.[19]
15. The aggregate installed thermal storage capacity is contingent upon the technical and economic conditions of the discrete industrial sites. The specific capacity at each site is influenced by the local heat consumers, their process requirements, and the required temperature levels. Typically, several consumers are situated together at a particular location (e.g., an industrial park), and are collectively supplied by a service provider. Each site would optimize its thermal storage size based on individual technical constraints, as well as storage and investment costs. This optimized capacity is available as a fixed value to the electricity supply system for demand response purposes. We assume an upper bound for realizing thermal storage capacity of $30\,\%$ of the process heat requirement by the year 2030 ($421.2\,\mathrm{GWh_{th}}$).
16. The operating hours of the process heat amount to $8760\,\mathrm{h}$.
17. The thermal energy storage is a packed-bed variety with a storage efficiency of $90\,\%$.[20]
18. The maximum thermal storage duration is 72 h.

The electrical power requirement for the heat supply of the high-temperature heat pump $P_{el,HP}^{PH}$ and the resistance heater $P_{el,RH}^{PH}$ was calculated using equation 2 and 3:

$$P_{el,HP}^{PH} = \frac{Q_{20-160\,^{\circ}\mathrm{C}}^{\mathrm{PH}}}{COP} \tag{2}$$

$$P_{\mathrm{el,RH}}^{\mathrm{PH}} = \frac{Q_{160-500\,^{\circ}\mathrm{C}}^{\mathrm{PH}}}{\eta_{\mathrm{RH}}} \tag{3}$$

with Q^{PH} being the required thermal energy for process heating, COP the average coefficient of performance (COP) of the high temperature heat-pump and η_{RH} the efficiency factor of the resistance heater.

The maximum available storage capacity in process heat $P_{\mathrm{el,S}}^{\mathrm{PH}}$ is determined by the demand for installed electrical power for process heat under assumptions 15 to 18, as well as by the maximum heat storage duration τ^{S}, and by the efficiency of the thermal storage η_{HS}:

$$P_{\mathrm{el,S}}^{\mathrm{PH}} = 0.3 \cdot \frac{Q^{\mathrm{PH}}}{\tau^{\mathrm{S}} \cdot \eta_{\mathrm{HS}}} \tag{4}$$

To reflect the cost of load flexibility for process heat, investment costs and flexibility costs due to heat

losses over the storage period are considered. This is done under the following assumptions:

19. The realization of load flexibility requires an overcapacity for the process heating of $70\,\%$ of the thermal storage capacity. We consider that the specific investment costs for this overcapacity $I_{\mathrm{EB}}^{\mathrm{PH}}$ amount to $80\,\mathrm{euro/kWh_{th}}$ for a resistance heater.[19] Any excess capacity in addition to this can be met by existing capacities, which are typically dimensioned at a higher level to cope with peak loads.
20. The investment costs of the fixed bed thermal storage are $40\,\mathrm{euro/kWh_{th}}$.[21]
21. The storage losses as a percentage of capacity per day are $3\,\%$.[21]

Given the efficiency of the thermal storage tank, and the efficiency factor of the resistance heater η_{RH}, as well as assumption 19 and 20, the specific investment costs $I_{\mathrm{DR}}^{\mathrm{PH}}$ in $\mathrm{euro/kWh_{el}}$ for load flexibilization of the process heat are calculated as follows:

$$I_{\mathrm{DR}}^{\mathrm{PH}} = \frac{I_{\mathrm{S}}^{\mathrm{PH}}}{\eta_{\mathrm{HS}} \cdot \eta_{\mathrm{RH}}} + \frac{I_{\mathrm{EB}}^{\mathrm{PH}} \cdot 0.7}{\eta_{\mathrm{RH}}} \tag{5}$$

S3. Further results

S3.1. Flexible demand capacities

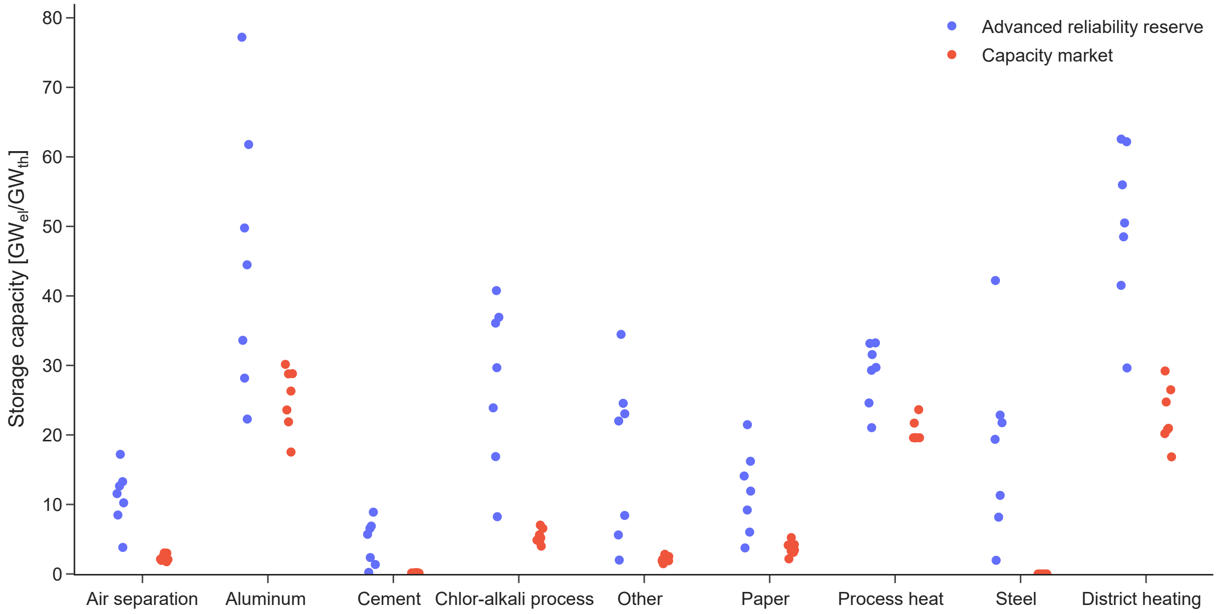

Notes: Storage capacity in $\mathrm{GWh_{el}}$ for all technologies except for district heating in $\mathrm{GWh_{th}}$. Generation and storage capacities (of technologies in the power sector) are fixed, while the capacities of the flexible demand technologies are optimized endogenously for different weather years.

Figure S2: Optimal investment into flexibility in different weather years (2008–2014), related to Figure 3

As shown by many previous studies, the choice of weather year strongly affects the results of optimization models. With fixed capacities in the power sector (generators and storage), the optimal capacity size of the demand-side flexibility options varies significantly depending on the assumed weather years (Figure S2). The reason is that different weather years exhibit different types and lengths of periods of scarce renewable energy supply, in combination with different demand patters, which in turns leads to different optimal model outcomes. Despite the variation between weather years, the impact of the centralized capacity market and the advanced reliability reserve on optimal demand-side flexibility capacities remains the same. The latter leads to more investment.

S3.2. Sensitivities with alternative activation prices

As discussed in the discussion section, the activation price is an important design parameter of the advanced reliability reserve. We illustrate how the main outcomes would change in our model setting under alternative activation prices that are $20\,\%$ lower or higher than assumed in the central scenario, i.e., $400\,\mathrm{euro/MWh_{el}}$ or $600\,\mathrm{euro/MWh_{el}}$.

If the activation price decreased to $400\,\mathrm{euro/MWh_{el}}$, the size of the reserve would grow from $35\,\mathrm{GW_{el}}$ to $37.0\,\mathrm{GW_{el}}$. For $600\,\mathrm{euro/MWh_{el}}$, it would conversely decrease to $34.6\,\mathrm{GW_{el}}$ (Figure S3). The capacity of open-cycle gas turbines respectively decreases or increases slightly, other capacities are not affected. With a lower activation price, open-cycle gas turbines earn lower infra-marginal rents whenever the reserve sets the wholesale market price, so their equilibrium capacity is lower. A higher activation price conversely leads to higher infra-marginal rents, which increases equilibrium investments in the wholesale market.

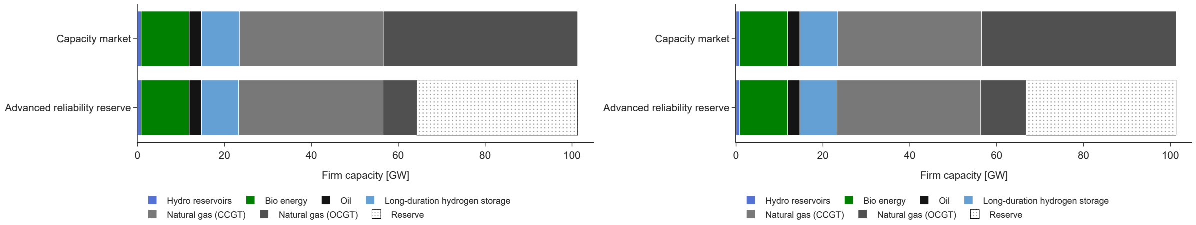

Figure S3: Installed firm capacity by technology for alternative activation prices of $400\,\mathrm{euro/MWh_{el}}$ (left) and $600\,\mathrm{euro/MWh_{el}}$ (right), related to Figure 1.

The variation in activation prices is directly visible in the resulting price-duration curves (Figure S4). An activation price of $400\,\mathrm{euro/MWh_{el}}$ not only effectively sets a cap on wholesale market prices at this level, but high prices are also spread over more hours than in the standard setup. Conversely, an activation price of $600\,\mathrm{euro/MWh_{el}}$ leads to respectively higher peak prices, but these occur in fewer hours. These changes in the duration result from interactions with demand-side flexibility technologies, which are shown next.

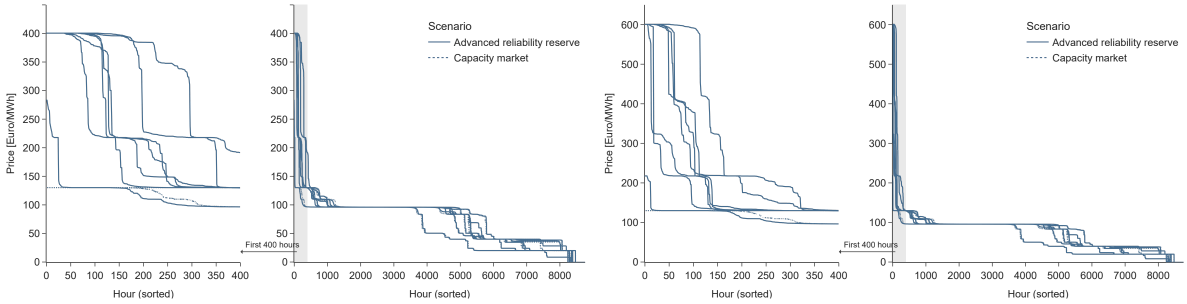

Figure S4: Wholesale market price-duration curves for different weather years (2008 – 2014) and activation prices of $400\,\mathrm{euro/MWh_{el}}$ (left) and $600\,\mathrm{euro/MWh_{el}}$ (right), related to Figure 2.

Investments in flexibility technologies in energy-intensive industries and in district heating storage slightly decrease with a lower activation price and increase with a higher activation price (Figure S5). Again, the reason for this is that wholesale market prices are effectively capped at a lower level in the former case and at a higher level in the latter, which results in lower or higher price differentials against which flexibility technologies can operate. However, the general results appear rather robust against the 20 % variation in the activation price parameter assumed here.

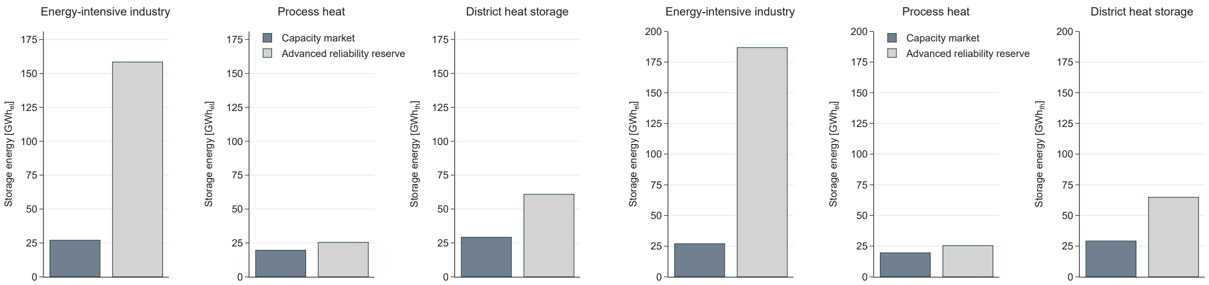

Figure S5: Storage capacities for enabling demand-side flexibility for alternative activation prices of $400\,\mathrm{euro/MWh_{el}}$ (left) and $600\,\mathrm{euro/MWh_{el}}$ (right), related to Figure 3.

For lower activation prices, the reserve is not only larger, but it is also activated more often. For higher activation prices, the opposite is true (Figure S6). Depending on the weather year, the reserve is used more than 100 hours per year in the $400\,\mathrm{euro/MWh_{el}}$ case, and only a good 50 hours with an activation price of $600\,\mathrm{euro/MWh_{el}}$. These results reflect the differences in the price-duration curves discussed above.

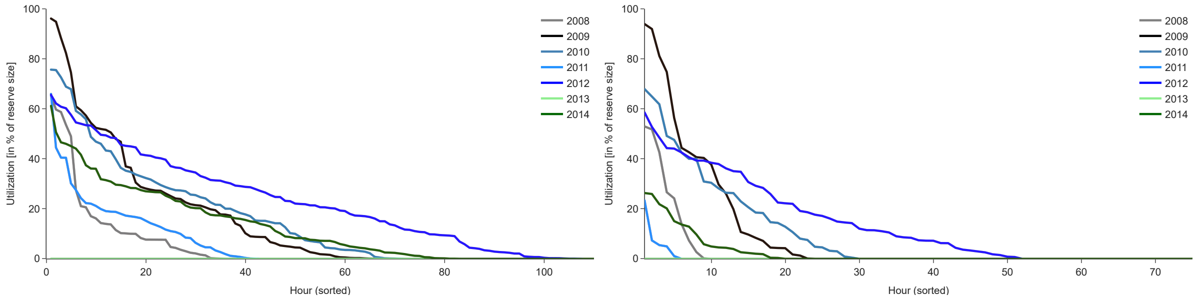

Figure S6: Operating hours of the advanced reliability reserve in different weather years for alternative activation prices of $400\,\mathrm{euro/MWh_{el}}$ (left) and $600\,\mathrm{euro/MWh_{el}}$ (right), related to Figure 4.

Overall costs hardly change with the 20 % variation in the reserve activation price. For the lower activation price sensitivity, both average wholesale prices and the capacity levy tend to increase slightly. The first is driven by a larger number of high prices (despite a lower price cap), as shown above, and the latter by a larger overall reserve capacity. Combining both effects, the average supply costs of the reliability reserve increase to $78.7\,\mathrm{euro/MWh_{el}}$ over all modeled weather years, compared to $77.6\,\mathrm{euro/MWh_{el}}$ in the central

scenario. For the higher activation price sensitivity, the opposite reasoning applies, and average supply costs slightly decrease to $77.5\,\mathrm{euro/MWh_{el}}$.

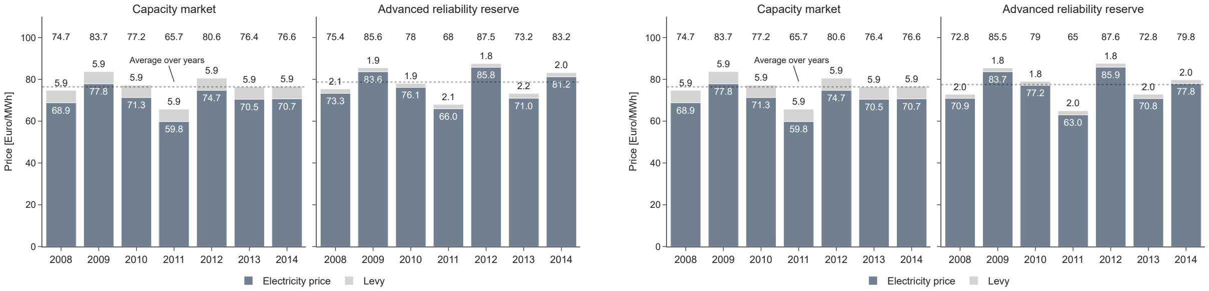

Figure S7: Average wholesale electricity prices and levies in different weather years (2008 – 2014) for alternative activation prices of $400\,\mathrm{euro/MWh_{el}}$ (left) and $600\,\mathrm{euro/MWh_{el}}$ (right), related to Figure 5.

References

[1] BAFA, Hintergrundinformationen zur Besonderen Ausgleichsregelung: Antragsverfahren 2021 für Begrenzung der EEG-Umlage 2022, Bundesamt für Wirtschaft und Ausfuhrkontrolle. Accessed: 2025-04-30 (2021).
URL `https://www.bafa.de/SharedDocs/Downloads/DE/Energie/bar_hintergrundinformationen.html`

[2] Boston Consulting Group, KLIMAPFADE 2.0 Ein Wirtschaftsprogramm für Klima und Zukunft, accessed: 2024-05-06 (2021).
URL `https://web-assets.bcg.com/58/57/2042392542079ff8c9ee2cb74278/klimapfade-study-german.pdf`

[3] DENA, dena-Netzstudie II. Integration erneuerbarer Energien in die deutsche Stromversorgung im Zeitraum 2015 – 2020 mit Ausblick 2025., accessed: 2025-04-08 (2010).
URL `https://www.dena.de/fileadmin/dena/Dokumente/Pdf/9106_Studie_dena-Netzstudie_II_deutsch.PDF`

[4] M. Paulus, F. Borggrefe, The potential of demand-side management in energy-intensive industries for electricity markets in Germany, Applied Energy 88 (2) (2011) 432–441. `doi:10.1016/j.apenergy.2010.03.017`.

[5] T. Langrock, S. Achner, C. Jungbluth, C. Marambio, A. Michels, P. Weinhard, Potentiale regelbarer Lasten in einem Energieversorgungssystem mit wachsendem Anteil erneuerbarer Energien, accessed:

2025-04-09 (2015).
URL http://www.umweltbundesamt.de/publikationen/potentiale-regelbarer-lasten-in-einem

[6] M. Steurer, Analyse von Demand Side Integration im Hinblick auf eine effiziente und umweltfreundliche Energieversorgung, Ph.D. thesis, Universität Stuttgart (2017). doi:10.18419/opus-9181.

[7] K. Helin, A. Käki, B. Zakeri, R. Lahdelma, S. Syri, Economic potential of industrial demand side management in pulp and paper industry, Energy 141 (2017) 1681–1694. doi:10.1016/j.energy.2017.11.075.

[8] VDE, Ein notwendiger Baustein der Energiewende: Demand Side Integration: Studie der Energietechnischen Gesellschaft im VDE (ETG), VDE Verband der Elektrotechnik Elektronik Informationstechnik e.V. (2012).

[9] F. Klaucke, R. Müller, M. Hofmann, J. Weigert, P. Fischer, S. Vomberg, G. Tsatsaronis, J.-U. Repke, Chlor-alkali Process with Subsequent Polyvinyl Chloride Production-Cost Analysis and Economic Evaluation of Demand Response, Industrial & Engineering Chemistry Research 62 (19) (2023) 7336–7351. doi:10.1021/acs.iecr.2c04188.

[10] F. Klaucke, T. Karsten, F. Holtrup, E. Esche, T. Morosuk, G. Tsatsaronis, J.-U. Repke, Demand Response Potentials for the Chemical Industry, Chemie Ingenieur Technik 89 (9) (2017) 1133–1141. doi:10.1002/cite.201600073.

[11] A. Kollmann, M. Schmidthaler, C. Elbe, E. Schmautzer, A. Kraussler, H. Steinmüller, F. Frank, L. Rebhandl, LoadShift: Lastverschiebung in Haushalt, Industrie, Gewerbe und kommunaler Infrastruktur Potenzialanalyse für Smart Grids, accessed: 2025-01-13 (2015).
URL https://nachhaltigwirtschaften.at/resources/e2050_pdf/reports/endbericht_201507b_loadshift_rahmenbedingungen.pdf

[12] C. Hoffmann, J. Hübner, F. Klaucke, N. Milojević, R. Müller, M. Neumann, J. Weigert, E. Esche, M. Hofmann, J.-U. Repke, R. Schomäcker, P. Strasser, G. Tsatsaronis, Assessing the Realizable Flexibility Potential of Electrochemical Processes, Industrial & Engineering Chemistry Research 60 (37) (2021) 13637–13660. doi:10.1021/acs.iecr.1c01360.

[13] F. Klaucke, C. Hoffmann, M. Hofmann, G. Tsatsaronis, Impact of the chlorine value chain on the demand

response potential of the chloralkali process, Applied Energy 276 (2020) 115366. doi:10.1016/j.apenergy.2020.115366.

[14] J.-C. Hourcade, D. Demailly, K. Neuhoff, M. Sato, Climate Strategies Report: Differentiation and Dynamics of EU ETS Industrial, accessed: 2025-04-20 (2007).
URL http://www.eprg.group.cam.ac.uk/wp-content/uploads/2008/11/competitiveness_final_report.pdf

[15] stahlpreise.eu, Stahlpreis, accessed: 2024-06-21 (2024).
URL https://www.stahlpreise.eu/

[16] finanzen.net GmbH, Aluminiumpreis, accessed: 2024-06-21 (2024).
URL https://www.finanzen.net/rohstoffe/aluminiumpreis

[17] GTAI, Der Bausektor legt 2024 einen Zahn zu, accessed: 2024-06-21 (2024).
URL https://www.gtai.de/de/trade/usa-wirtschaft/bauwirtschaft

[18] A. Kemmler, S. Straßburg, F. Seefeldt, N. Anders, C. Rohde, T. Fleiter, A. Aydemir, H. Kleeberger, L. Hardi, B. Geiger, Datenbasis zur Bewertung von Energieeffizienzmaßnahmen in der Zeitreihe 2005 – 2014, accessed: 2025-04-08 (2017).
URL https://www.umweltbundesamt.de/sites/default/files/medien/1968/publikationen/2017-01-09_cc_01-2017_endbericht-datenbasis-energieeffizienz.pdf

[19] T. Fleiter, M. Rehfeldt, S. Hirzel, L. Neusel, A. Aydemir, C. Schwotzer, F. Kaiser, C. Gondorf, J. Hauch, J. Hof, L. Sankowski, Langhorst, Moritz, CO2-neutrale Prozesswärmeerzeugung: Umbau des industriellen Anlagenparks im Rahmen der Energiewende: Ermittlung des aktuellen SdT und des weiteren Handlungsbedarfs zum Einsatz strombasierter Prozesswärmeanlagen, accessed: 2024-05-06 (2023).
URL https://www.umweltbundesamt.de/sites/default/files/medien/11850/publikationen/161_2023_texte_prozesswaermepumpen_0.pdf

[20] A. Profaiser, W. Saw, G. J. Nathan, P. Ingenhoven, Bottom-up estimates of the cost of supplying high-temperature industrial process heat from intermittent renewable electricity and thermal energy storage in Australia, Processes 10 (6) (2022) 1070. doi:10.3390/pr10061070.

[21] K. Arnold, F. Ausfelder, P. Bartsch, T. Bayer, C. Dannert, C. Dufter, H. E. Dura, J. Fischer, T. Fischer, B. Fleischmann, M. Frank, K. Ganz, A. Gruber, A. Guminski, G. Holtz, S. S. Hosseinioun, T. Hübner, T. Kern, B. Kleinertz, O. Krause, S. Lindner, S. Möhring, R. Pietruck, A. Queck, J. C. Richstein, H. Rosemann, S. Rösch, J. Ruppert, S. Seemann, A. Taubitz, K. Treiber, F. Veitengruber, S. von Roon, H. Wuthnow, D. Zinsmeister, S. Zunft, Flexibilitätsoptionen in der Grundstoffindustrie II: Analysen, Technologien, Beispiele : Bericht des AP V.6 "Flexibilitätsoptionen und Perspektiven in der Grundstoffindustrie" im Kopernikus-Projekt "SynErgie - Synchronisierte und energieadaptive Produktionstechnik zur flexiblen Ausrichtung von Industrieprozessen auf eine fluktuierende Energieversorgung", 1st Edition, DECHEMA Gesellschaft für Chemische Technik und Biotechnologie e.V, Frankfurt am Main, 2019.
URL `https://dechema.de/dechema_media/Downloads/Positionspapiere/2019_Kopernikus_Flexoptionen_Band%2BII_kompl.pdf`
